# On the Internal Use of the Spirit of Turpentine in Incarcerated Hernia

**Published:** 1830-03

**Authors:** Thomas Sewall

**Affiliations:** Professor of Anatomy and Physiology in the Columbian College, District of Columbia.


### TURPENTINE IN HERNIA.

*On the internal Use of the Spirit of Turpentine in Incarcerated Hernia.* By Thomas Sewall, m.d. Professor of Anatomy and Physiology in the Columbian College, District of Columbia.

Some time since, I was informed by Dr. M'Williams, a highly respectable physician of this city, that he had recently met with two cases of incarcerated hernia, in which the spirit of turpentine, exhibited in large doses internally, was successful in effecting a reduction of the protruded part, after all other means had failed. The circumstances of these cases were such as to inspire me with some confidence in the powers of the turpentine, and induce me to determine on its application as soon as an opportunity should present. It was not long after that the following case of incarcerated scrotal hernia came under my charge.

Early on Sunday morning, I was called to visit a labouring man, by the name of Penn, a brickmaker by trade, a short, robust person, of about twenty-five years of age. He had enjoyed perfect health, with the exception of an entero-scrotal hernia, under which he had laboured for a number of years, and for which he had worn a truss.

On my arrival, I found that he had been in a state of great suffering during the night, and that he was still affected with intense pain and high fever. From inquiry I learned that, while at work at his usual occupation, the day previous, the intestine escaped through the abdominal ring, and descended into the scrotum, and that all the efforts which he could make to reduce it were ineffectual. On examining the scrotum, I found it greatly distended, hard, and tender to the touch.

I first attempted a reduction of the bowel by taxis; but, as my exertions were unavailing, I bled him largely, and then renewed my exertions, but without success. I then gave him two ounces of the spirit of turpentine, and instructed my pupils, who remained with him, to repeat the same dose every hour, till eight ounces were taken, or some sensible effect produced. Soon after I left him, a profuse sweat took place, and he fell into a tranquil sleep. In about two hours, the hernial tumor became soft and yielding, and spontaneously retired from the scrotum. On repeating my visit in the middle of the day, I found he had taken about six ounces of the turpentine, and without experiencing any inconvenience from it. He was still sleeping, and entirely relieved. The next day he was at work in the brickyard, and with no other complaint than that of a slight looseness of the bowels, and a scalding sensation in the rectum in passing his stools. No strangury was produced.

Although it requires the experience derived from many cases to entitle a new remedy to confidence, the beneficial effects of the turpentine were so obvious and striking in the above cases, that I have thought proper to call the attention of the profession to its further trial.*

* Ibid.

